# Bioinformatics Analysis Reveals MicroRNA-193a-3p Regulates ACTG2 to Control Phenotype Switch in Human Vascular Smooth Muscle Cells

**DOI:** 10.3389/fgene.2020.572707

**Published:** 2021-01-12

**Authors:** Weitie Wang, Yong Wang, Hulin Piao, Bo Li, Zhicheng Zhu, Dan Li, Tiance Wang, Kexiang Liu

**Affiliations:** Department of Cardiovascular Surgery of the Second Hospital of Jilin University, The Second Hospital of Jilin University, Changchun, China

**Keywords:** mRNA, miRNA, vascular disease, aortic dissection, cardiovascular

## Abstract

Aortic dissection (AD) is among the most fatal cardiovascular diseases. However, the pathogenesis of AD remains poorly understood. This study aims to integrate the microRNAs (miRNA) and mRNA profiles and use bioinformatics analyses with techniques in molecular biology to delineate the potential mechanisms involved in the development of AD. We used the human miRNA and mRNA microarray datasets GSE98770, GSE52093, and GEO2R, Venn diagram analysis, gene ontology, and protein–protein interaction networks to identify target miRNAs and mRNAs involved in AD. RNA interference, western blotting, and luciferase reporter assays were performed to validate the candidate miRNAs and mRNAs in AD tissues and human vascular smooth muscle cells (VSMCs). Furthermore, we studied vascular smooth muscle contraction in AD. *In silico* analyses revealed that miR-193a-3p and ACTG2 were key players in the pathogenesis of AD. miR-193a-3p was upregulated in the AD tissues. We also found that biomarkers for the contractile phenotype in VSMCs were downregulated in AD tissues. Overexpression and depletion of miR-193a-3p enhanced and suppressed VSMC proliferation and migration, respectively. Dual luciferase reporter assays confirmed that ACTG2 was a target of miR-193a-3p. ACTG2 was also downregulated in human AD tissues and VMSCs overexpressing miR-193a-3p. Taken together, miR-193a-3p may be a novel regulator of phenotypic switching in VSMCs and the miR-193a-3p/ACTG2 axis may serve as a promising diagnostic biomarker and therapeutic candidate for AD.

## Introduction

Type A aortic dissection (AD) is a fatal cardiovascular disease associated with high morbidity and mortality, and requires a complex treatment regimen (Elsayed et al., 2017; Silaschi et al., 2017). Vascular smooth muscle cells (VSMCs) are involved in vascular function and have been implicated in the pathogenesis of AD (Zhang et al., 2016; Wei et al., 2017). However, the precise mechanisms involved in AD remain to be fully understood. Therefore, it is important to delineate the roles of VSMCs in AD and phenotypic plasticity. This will help identify new modes of treatment, especially targeted drug therapy. Owing to advances in sequencing technology, differential genes expression between normal and damaged tissues have been widely used to identify candidate pathogenic genes (Wang et al., 2019a). Bioinformatics tools can analyze such high-throughput data, while omitting “junk” data to provide reliable analyses.

Vascular smooth muscle cells phenotypic remodeling is mainly relevant with intima-media thickening which acts important roles during the pathological progression for vascular disease, such as AD (Gomez and Owens, 2012). Although the pathogenesis of AD remains unclear, VSMCs phenotypic remodeling alternating from contractile to synthetic in response to stimulation are essential in AD, which regulates vascular remodeling. Contractile VSMCs generally demonstrate reduced ability in proliferation and migration, whereas synthetic VSMCs present enhanced viability in proliferation and migration (Alexander and Owens, 2012). Downregulation of differentiation markers such as SMA, SM22, and MYH11 always happen in synthetic VSMCs. Therefore, detection of differentiation markers suggests the VSMCs phenotypic remodeling. Numerous factors including growth factor and cell adhesion molecules promote the phenotypic switch to synthetic VSMCs and enhance cell proliferation and migration (Gomez and Owens, 2012). However, the molecular mechanisms between VSMC phenotypic switch and AD remain unclear.

MicroRNAs (miRNAs) are non-coding RNAs that function by binding to target mRNAs. miRNAs regulate various cellular functions, including proliferation and migration, and phenotypic switch in VSMCs (Tang et al., 2017; Li et al., 2019). Therefore, it is imperative to understand the importance of miRNAs and their targets in VSMC function in patients with AD.

In this study, we combined bioinformatics analysis with techniques in molecular biology to elucidate key miRNAs and mRNAs involved in AD. GEO2R analysis, target gene prediction, gene ontology (GO) and pathway analysis, Venn diagrams, and protein–protein interaction (PPI) networks were used to assess the roles of miR-193a-3p/ACTG2 as candidates associated with phenotypic switching in VSMCs. Our study presented that *in vitro* cell culture-based experiments revealed miR-193a-3p/ACTG2 participated in the development of AD.

## Materials and Methods

### Datasets and Workflow

**FIGURE 1:**
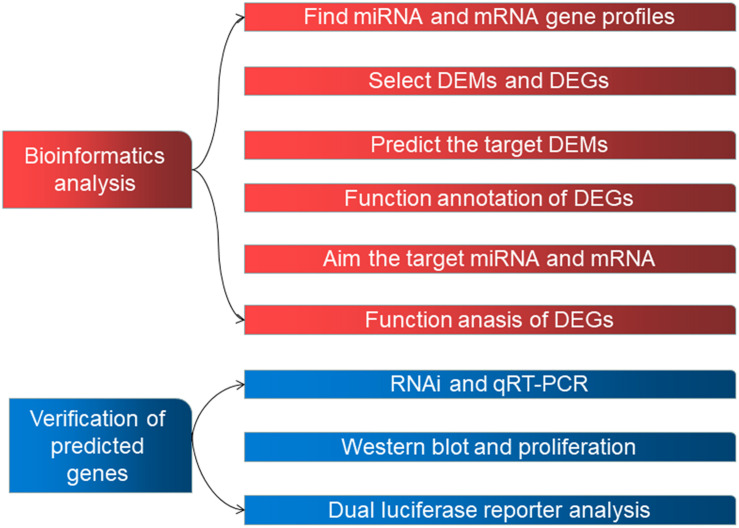
The workflow used in this study.

Human datasets GSE98770 (GPL14550) and GSE52093 (GPL10558) were obtained from the GEO database. We analyzed 11 samples from GSE98770 (six AD and five normal ascending tissues) and 12 samples from GSE52093 (seven AD and five normal ascending tissues). Figure 1 shows the workflow used in the study.

### Analyzing the Differential Expression of RNAs

GEO2R with R software was used to determine the gene expression profiles of AD and normal control (NC) samples and identify genes that were differentially expressed. Data from normal and AD samples were arranged in order before using the R software. *P* < 0.05 and | Log fold change| ≥ 1.0 were used as the screening thresholds for datasets GSE98770 and GSE52093.

### Bioinformatic Analysis

All differentially expressed genes (DEGs) were analyzed using the DAVID online tool that included GO and Kyoto Encyclopedia of Genes and Genomes (KEGG) functional enrichment. The top pathways associated with the DEGs were further analyzed. We had previously determined the mRNA profiles for six AD and normal tissues each (Wang et al., 2019a) that were used to compare within dataset GSE52093 to identify the DEGs.

### Target mRNA Identification and Generation of the PPI Network

We generated the PPI network using geneMANIA^1^ for the DEGs associated with vascular smooth muscle contraction in dataset GSE52093 and our mRNA profiles. A confidence score >0.4 was considered significant.

### Prediction of Differentially Expressed miRNAs (DEMs)

All miRNAs expressed in GSE52093 and the predicted mRNA profiles correlating with vascular smooth muscle contraction were selected to predict the DEMs using TargetScan.^2^

### Venn Diagram Analysis

Differentially expressed miRNAs from the GSE98770 dataset and miRNAs predicted in this study were analyzed using Venn diagrams generated by using FunRich (Zhang and Wang, 2019).

### Human Aortic Samples

This study was conducted in accordance with the Declaration of Helsinki and was approved by the Ethical Committee at Jilin University (IRB:2019018). All patients agreed to participate in this study and provided written informed consent. Human ascending aorta specimens were acquired from AD patients (*n* = 10) and ischemic heart disease patients (*n* = 10) during surgery (Table 1). All AD patients were diagnosed using computed tomography angiography and patients with hereditary disease were excluded.

**TABLE 1:** Information of aortic dissection and ischemic heart disease patients.

	AD group	control group
		
	(*n* = 10)	(*n* = 10)
Age (years old)	48.3 ± 7.31	58.22 ± 5.91
Male	6(60.00%)	6(60.00%)
Maximal diameter, cm	5.81 ± 2.09	2.98 ± 0.34
Smoking	2(20.00%)	6(60.00%)
NYHA class III-IV	1(10.00%)	4(20.00%)
Hypertension	7(70.00%)	6(60.00%)
Diabetes mellitus	0(00.00%)	6(60.00%)
Chronic renal dysfunction	0	0

*AD, aortic dissection; NYHA, New York Heart Association.*

### Cell Culture

Human aortic VSMCs (Lonza, Walkersville, MD, United States) were cultured in smooth muscle culture medium (SMCM) (America Sciencell) supplemented with 2% fetal bovine serum and 1% smooth muscle cell growth supplement at 37°C in a humidified 5% CO_2_ incubator. Cells used in this study were between passage number 5 and 7. VSMCs were serum starved (0.5% fetal bovine serum) for 24 h and stimulated using PDGF-BB.

### Quantitative Reverse Transcription-Polymerase Chain Reaction

Total RNA was extracted using TRIzol (Invitrogen) according to the protocol provided. After measuring RNA concentration at 260 nm, the total RNA was reverse transcribed to cDNA (Takara Bio Inc., Japan) to analyze the mRNA and miRNA content in samples. Table 2 lists all the primers used in this study. Glyceraldehyde-3-phosphate dehydrogenase (GAPDH) and U6 were used as endogenous controls. We calculated relative gene expression using the 2^–ΔΔCt^ method.

**TABLE 2:** The primers in this work.

Gene name	Forward primer	Reverse primer
miR-193a-3p	ATGCTCAAACTGGCCTACAAG	TATGGTTGTTCTGCTCTCT GTCTC
ACTG2	GCGTGTAGCACCTGAAGAG	GAATGGCGACGTACATGGCA
SMA	GCGTGGCTATTCCTTCGTTA	ATGAAGGATGGCTGGAACAG
Calponin	AGCTAAGAGAAGGGCGGAAC	CATCTGCAGGCTGACATTGA
SM22a	AACAGCCTGTACCCTGATGG	CGGTAGTGCCCATCATTCTT
MMP-2	ACCCATTTACACCTACACCAAG	TGTTTGCAGATCTCAGGAGTG
MMP-9	CGAACTTTGACAGCGACAAG	CACTGAGGAATGATCTAAGCCC
MYH11	TGGAACTTCATCGACTTTGGG	ACAGCTTCTCCACGAAAGAC
U6	GCGCGTCGTGAAGCGTTC	GTGCAGGGTCCGAGGT
GAPDH	CGGACCAATACGACCAAATCCG	AGCCACATCGCTCAGACACC

*miR-193, microRNA-193; ACTG2, actin gamma 2; SMA, smooth muscle – actin; SM22, smooth muscle 22; MYH11, smooth muscle myosin heavy chain.*

### VSMC Transfection

The miR-193a-3p mimic and inhibitor were designed by GenePharma (Shanghai, China). VSMCs were transfected with 250 pmol of the mimic or inhibitor and 2 μl Transfection Reagent (TransDetect, Beijing, China) for ∼5 h. Cellular miRNA expression were analyzed 48 h after transfection.

### Western Blot Analysis

Total protein content of the cell was separated using sodium dodecyl sulfate-polyacrylamide gel electrophoresis and transferred to nitrocellulose membranes. Blots were blocked using 5% non-fat milk supplemented with 0.1% Tween 20. Subsequently, the blots were incubated with primary antibodies against ACTG2 (1:500, bioss), SM22a (1:500, Proteintech), SMA (1:800, bioss), calponin (1: 2,000, bioss), MYH (1:1,000, Proteintech), MMP-2 (1:500, abcam), and MMP-9 (1:500, abcam) followed by incubation with secondary antibodies. The images were analyzed using ImageJ.

### Dual Luciferase Reporter Assay

Wild or mutant 3′-untranslated region (UTR) of ACTG2 were cloned into pGL6-miR (Beyotime, China). We seeded 2 × 10^4^ HEK293T cells onto six-well plates for 24 h before transfection. The luciferase kit (Beyotime) was used after transfection (pGL6-ACTG2-wt and pGL6-ACTG2-mut) using Lipofectamine 2000 (Invitrogen, Life Technologies). Luciferase activity was measured at 560 nm. Signal from Renilla luciferase was normalized to that from firefly luciferase.

### Cell Proliferation Assay

We seeded ∼3 × 10^4^ cells VSMC in each well of a 96-well plate for 15–20 h before transfection. Cells were then transfected with the miR-193a-3p mimic and inhibitor and incubated for 20 h. Subsequently, the cells were incubated with medium containing the Cell Counting Kit (CCK) Solution (TransDetect, Beijing, China) for 4 h following which we measured absorbance at 450 nm.

### Wound Healing Assay

We seeded 2.5 × 10^5^ cells into each well of a six-well plate and transfected them with miR-193a-3p. After starving for 48 h, we generated a linear wound using a 200-μl tip. Cells were then incubated in medium for 48 and 72 h. Finally, cells that migrated into the wounded area were counted.

### Statistical Analysis

Continuous data are expressed as mean ± standard deviation. One-way analysis of variance (ANOVA) was performed between-group differences and multiple groups. Comparisons among multiple groups were performed using one-way ANOVAs, followed by *post hoc* Tukey tests. Relative expression of RT-qPCR was calculated using the ΔΔCq method.

## Results

### DEGs in AD

**FIGURE 2:**
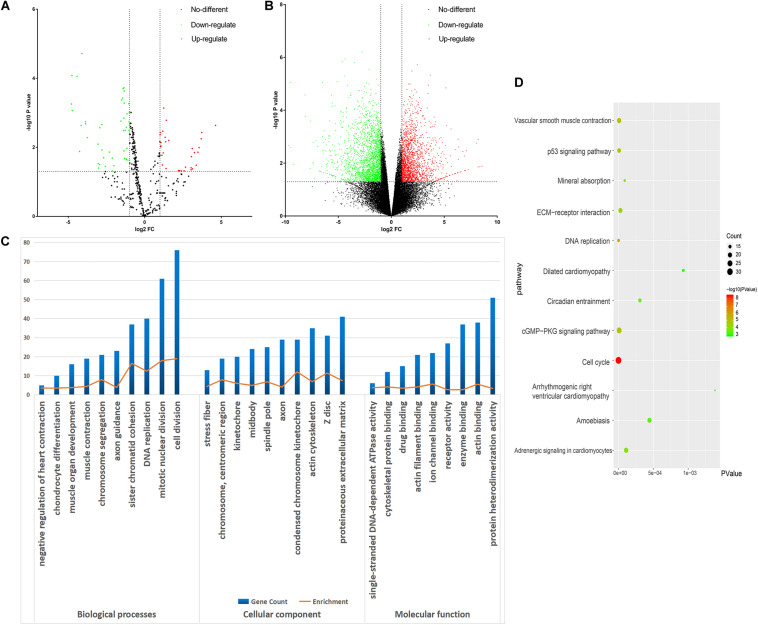
Bioinformatic analysis of the differentially expressed genes (DEGs). **(A)** Volcano plot of the miRNA profiles. **(B)** Volcano plot for mRNA expression. We used green and red to represent decrease and increase in expression, respectively. **(C)** Gene Ontology analysis of the DEGs. **(D)** Kyoto Encyclopedia of Genes and Genomes analysis of the DEGs. Gradual color means P value and the size means gene number.

All the raw data were first analyzed using the GEO2R tool. From the GSE98770 dataset, we identified 34 and 47 upregulated and downregulated miRNAs, respectively. From the GSE52093 dataset, we identified 1,409 and 1,235 upregulated and downregulated mRNAs, respectively. The volcano plots showed the DEMs (Figure 2A) and DEGs (Figure 2B) in the AD and NC samples.

### GO and KEGG Pathway Analysis

DAVID was used to functionally analyze the DEGs. The downregulated mRNAs correlated with muscle contraction and muscle organ development in AD, suggesting that downregulated genes primarily localized to the aortic media. Upregulated DEGs were involved in cell division and mitotic nuclear division, indicating the importance of cell division in AD (Table 3 and Figure 2C).

**TABLE 3:** Enriched analysis.

Expression	Category	Term	Description	Gene count	*P*-value
UP-DEGs	BP	cell division	GO:0051301	76	1.31E-19
	BP	mitotic nuclear division	GO:0007067	61	1.36E-18
	BP	sister chromatid cohesion	GO:0007062	37	3.82E-17
	BP	DNA replication	GO:0006260	40	4.61E-13
	BP	chromosome segregation	GO:0007059	21	1.29E-08
	CC	condensed chromosome kinetochore	GO:0000777	29	1.02E-12
	CC	chromosome, centromeric region	GO:0000775	19	1.61E-08
	CC	spindle pole	GO:0000922	25	1.73E-07
	CC	cytosol	GO:0005829	290	3.39E-07
	CC	membrane	GO:0016020	204	6.67E-07
	MF	protein binding	GO:0005515	643	2.32E-04
	MF	single-stranded DNA-dependent ATPase activity	GO:0043142	6	2.46E-04
	MF	drug binding	GO:0008144	15	4.36E-04
	MF	protein heterodimerization activity	GO:0046982	51	5.44E-04
	MF	ATP binding	GO:0005524	130	0.001165931
DOWN-DEGs	BP	muscle contraction	GO:0006936	19	5.16E-05
	BP	axon guidance	GO:0007411	23	1.69E-04
	BP	muscle organ development	GO:0007517	16	2.09E-04
	BP	chondrocyte differentiation	GO:0002062	10	3.58E-04
	BP	negative regulation of heart contraction	GO:0045822	5	3.67E-04
	CC	Z disc	GO:0030018	31	4.67E-12
	CC	proteinaceous extracellular matrix	GO:0005578	41	4.59E-08
	CC	actin cytoskeleton	GO:0015629	35	1.60E-07
	CC	plasma membrane	GO:0005886	296	4.28E-05
	CC	stress fiber	GO:0001725	13	5.30E-05
	MF	calcium ion binding	GO:0005509	78	2.79E-07
	MF	ion channel binding	GO:0044325	22	2.68E-06
	MF	actin binding	GO:0003779	38	3.43E-06
	MF	cytoskeletal protein binding	GO:0008092	12	9.05E-05
	MF	actin filament binding	GO:0051015	21	9.99E-05

Kyoto Encyclopedia of Genes and Genomes analysis revealed that the downregulated and upregulated genes were involved in vascular smooth muscle contraction and cell cycle, respectively (Figure 2D). Thus, GO and KEGG analyses highlighted the importance of vascular smooth muscle contraction in the aortic media during AD.

### PPI Network

**FIGURE 3:**
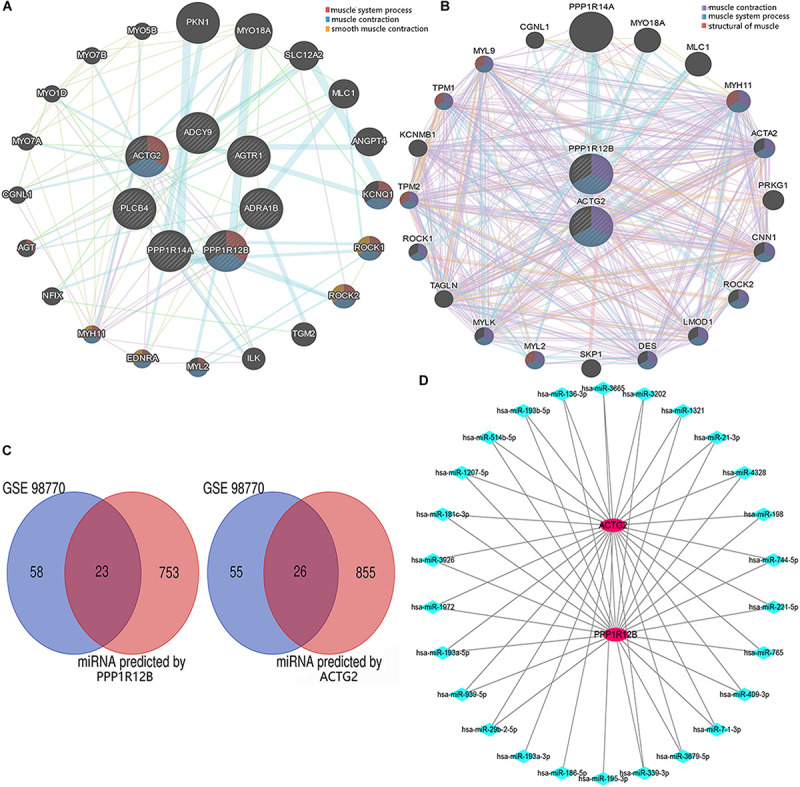
The interactome of DEGs. **(A)** Protein–protein interaction networks of the DEGs associated with the eight genes (MYH11, EDNRA, MYL2, ROCK2, ROCK1, and KCNQ1) relevant with vascular smooth muscle contraction pathway. **(B)** Protein–protein interaction networks of DEGs (ACTG2 and PPP1R12B) associated with vascular smooth muscle contraction. **(C)** Venn diagram for the DEGs (including PPP1R12B and ACTG2) in the GSE98770 dataset. **(D)** The miRNA–mRNA network. There were 25 and 2 differentially expressed miRNAs and mRNAs, respectively that were associated with vascular smooth muscle contraction. The red circles and blue diamonds represent the miRNAs and mRNAs, respectively.

We generated a PPI network with genes related to the vascular smooth muscle contraction pathway using GeneMANIA bio-informatic analysis. Results showed that 8 genes (ACTG2 and PPP1R12B predicted by DAVID tool. MYH11, EDNRA, MYL2, ROCK2, ROCK1, and KCNQ1 predicted by GeneMANIA) were involved in muscular system processes (FDR:1.40e-7, nine gene count), muscle contraction (FDR:1.26e-6, eight gene count), and smooth muscle contraction (FDR:1.65e-3, four gene count) (Figure 3A). Then ACTG2 and PPP1R12B were used for PPI network analysis by GeneMANIA, which also indicated changing muscle function was relevant in AD (Figure 3B). Thus, combined with PPI analyses and previous GO and KEGG analyses, ACTG2 and PPP1R12B may be crucial genes involved in AD.

### Target Gene Prediction and Validation

ACTG2 and PPP1R12B were used to predict potential target miRNAs by TargetScan. About 881 predicted miRNAs for ACTG2 and 776 predicted miRNAs for PPP1R12B (score ≥ 80) were collected and compared to 81 candidate miRNAs from GSE98770 to identify DEMs. Venn diagram analysis showed 49 DEMs (Figure 3C) that were common to data from GSE98770 and the miRNAs predicted in this study. A miRNA–mRNA network was constructed using Cytoscape (Figure 3D). miR-193a-3p was upregulated and among the top 3 predicted miRNAs with high score and binding sites for ACTG2. Furthermore, miR-193a-3p is crucial for cell proliferation. Therefore, miR-193a-3p may regulate VSMC proliferation.

### miR-193a-3p Regulates VSMC Phenotypes

**FIGURE 4:**
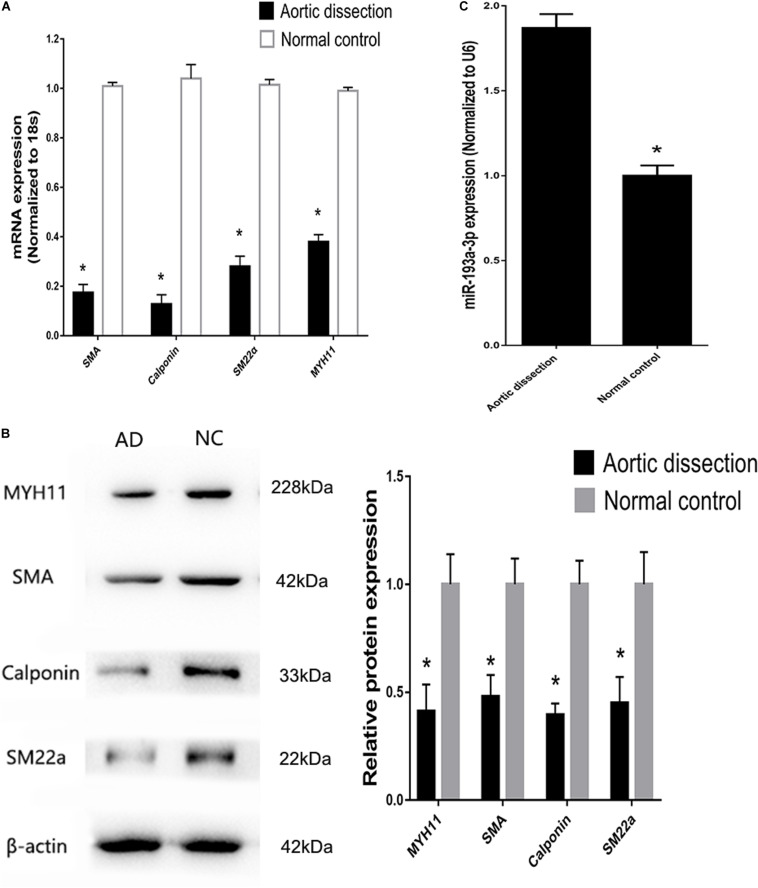
Increase of miR-193a-3p levels in tissues from patients with aortic dissection (AD). **(A)** Quantitative reverse transcription-polymerase chain reaction (qRT-PCR) showed a decrease in VSMC differentiation biomarkers in AD tissues (*n* = 10). *P < 0.05. **(B)** Western blot analysis showing downregulation of VSMC differentiation biomarkers in AD tissues (*n* = 3). *P < 0.05. **(C)** miR-193a-3p was upregulated in human AD tissues.

Tissues near the intimal tear isolated from patients with AD exhibited decreased expression of differentiation biomarkers (SMA, SM22, calponin, and MYH11) and contained highly proliferative VSMCs compared to those in tissues from healthy individuals (Figures 4A,B). At the meantime, miR-193a-3p also increased in the AD tissues (Figure 4C). These results highlight the involvement of miR-193a-3p in human VSMC function and phenotypic plasticity.

### Overexpression of miR-193a-3p in Proliferative VSMCs

**FIGURE 5:**
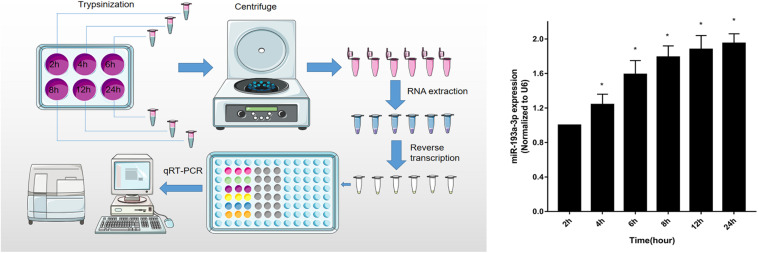
Upregulation of miR-193a-3p in proliferating VSMCs. qRT-PCR showed the time-dependent increase of miR-193a-3p in VSMCs (*n* = 5). *P < 0.05 compared to the 24 h cells.

To identify that miR-193a-3p is specifically involved in the regulation of VSMC, quantitative reverse transcription-polymerase chain reaction (qRT-PCR) was used to detect miR-193a-3p expression levels in VSMCs with increasing duration of proliferation (2, 4, 6, 8, 12, and 24 h). The qRT-PCR results showed that miR-193a-3p was upregulated in VSMCs with an increase in cell proliferation. As shown in Figure 5, miR-193a-3p was upregulated in a time-dependent manner together with an increase in VSMC proliferation.

### miR-193a-3p Regulates Smooth Muscle Biomarkers in VSMCs

**FIGURE 6:**
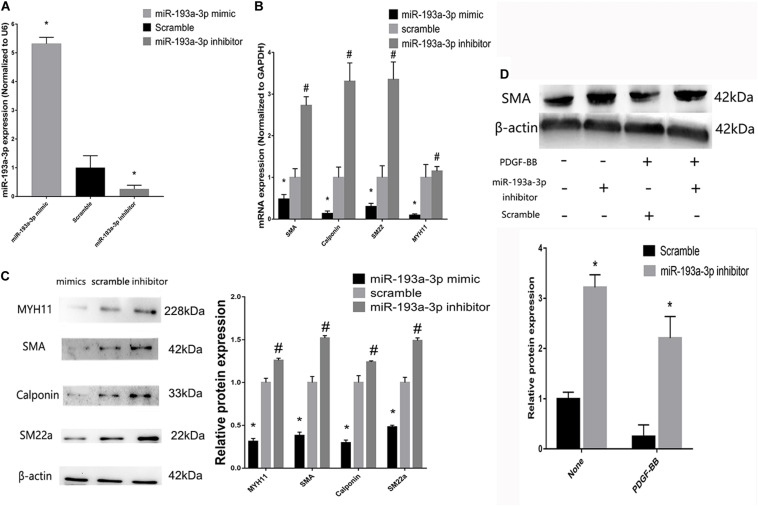
miR-193a-3p regulates smooth muscle biomarkers *in vitro*. **(A)** qRT-PCR showing the overexpression and downregulation of miR-193a-3p after transfection with the miR-193a-3p mimic and inhibitor, respectively (*n* = 10, *P ≤ 0.05 vs. Scramble). **(B)** miR-193a-3p inhibitor increased the expression of the differentiation biomarkers, whereas miR-193a-3p mimic downregulated the biomarkers (*n* = 10, *P ≤ 0.05 vs. Scramble, ^#^*P* < 0.05 vs. Scramble). **(C)** Western blots showing the decrease and increase in differentiation biomarker expression in miR-193a-3p inhibitor and mimic-transfected cells, respectively (*n* = 3, *P < 0.05 vs. Scramble, ^#^*P* ≤ 0.05 vs. Scramble). **(D)** Effect of miR-193a-3p on SMA expression in PDGF-BB stimulated VSMCs by densitometric analysis of SMA protein levels as measured by Western blot analysis (*n* = 5). *P < 0.05 vs. scramble with or without PDGF-BB treatment (20 ng/ml).

To investigate the role of miR-193a-3p in human aortic VSMC phenotype switch, we transiently transfected miR-193a-3p mimic and miR-193a-3p inhibitor into human aortic VSMCs. qRT-PCR was used to confirm the transfection efficiency of the miR-193a-3p mimic, inhibitor, and controls. Figure 6A showed that the miR-193a-3p mimic elevated miR-193a-3p levels, whereas miR-193a-3p inhibitor markedly reduced the endogenous levels of miR-193a-3p in VSMCs. Western blotting and qRT-PCR showed the decrease in biomarkers involved in VSMC differentiation, such as SMA, SM22a, calponin, and MYH11 (Figures 6B,C), whereas lower levels of miR-193a-3p were associated with upregulation of these biomarkers in human VSMCs. Western blot analysis also demonstrated that the SMA protein levels were dramatically increased after ectopic downregulation of miR-193a-3p under both basal and PDGF-BB-stimulated conditions. Thus, miR-193a-3p levels regulated VSMC differentiation.

### miR-193a-3p Regulates VSMC Proliferation and Migration

**FIGURE 7:**
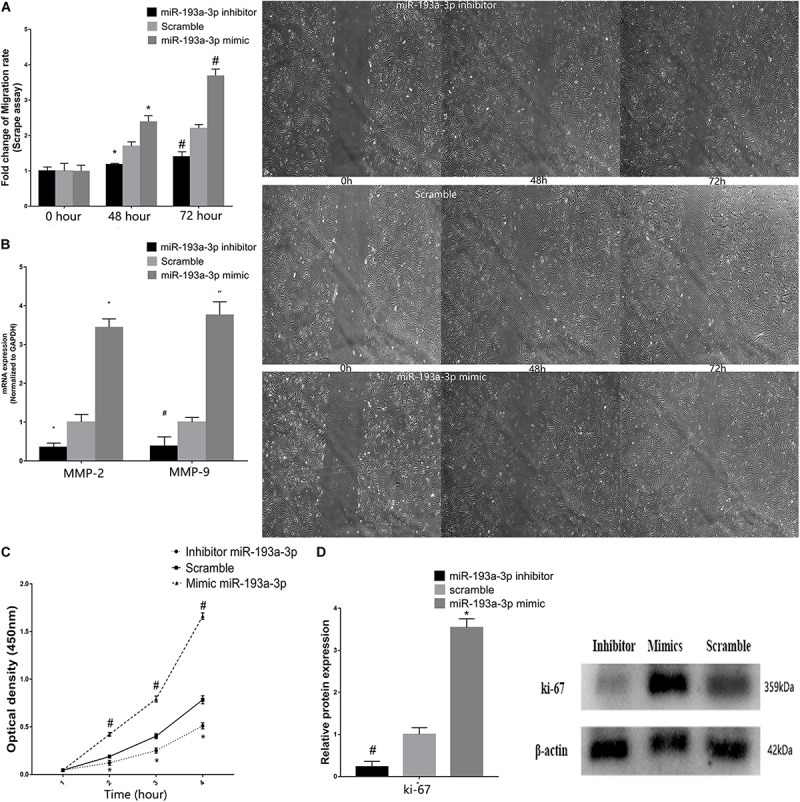
miR-193a-3p in VSMC proliferation and migration. **(A)** Scrape assay showing the reduction in the migration of human aortic VSMCs transfected with miR-193a-3p inhibitor. Cells transfected with the miR-193a-3p mimic exhibited enhanced cell migration (*P ≤ 0.05 vs. Scramble, ^#^*P* ≤ 0.05 vs. Scramble). **(B)** qRT-PCR showing the increase in mRNA levels of MMP-2 and MMP-9 in VSMCs transfected with the miR-193a-3p mimic. **(C)** The miR-193a-3p mimic enhanced VSMC proliferation as measured by the cell counting kit-8 (*n* = 10, *P < 0.05 vs. 1 h, ^#^*P* ≤ 0.05 vs. Scramble). **(D)** Western blots showing the upregulation of Ki-67 in cells transfected with the miR-193a-3p mimic (*P ≤ 0.05 vs. Scramble, ^#^*P* ≤ 0.05 vs. Scramble).

Vascular smooth muscle cells were transfected with miR-193a-3p mimic, inhibitor, or control. Subsequently, the scratch assay was used to evaluate the effect of miR-193a-3p on cell migration. Figure 7A showed the marked increase in migration in cells transfected with the miR-193a-3p mimic. In addition, we also detected the migration biomakers such as MMP-2 and MMP-9, which have been implicated in VSMC migration. MMP-2 and MMP-9 RNA levels were also higher in the miR-193a-3p mimic-transfected cells (Figure 7B). These results showed that high expression of miR-193a-3p promoted VSMC migration. As shown in Figure 7C, we assayed the effect of miR-193a-3p on VSMC proliferation using CCK-8. Cell proliferation was recorded at 1, 2, 3, and 4 h. Cells overexpressing miR-193a-3p exhibited increased proliferation as compared to the control cells. However, VSMCs depleted of miR-193a-3p showed reduced proliferation. Moreover, we also detected the proliferation biomaker (Ki-67). Western blots showed the upregulation of Ki-67 in miR-193a-3p-overexpressing VSMCs, whereas inhibition of miR-193a-3p in VSMCs downregulated Ki-67 (Figure 7D). Taken together, these results indicated that miR-193a-3p was a promoter of VSMC proliferation and migration.

### ACTG2 Is a Target of miR-193a-3p

**FIGURE 8:**
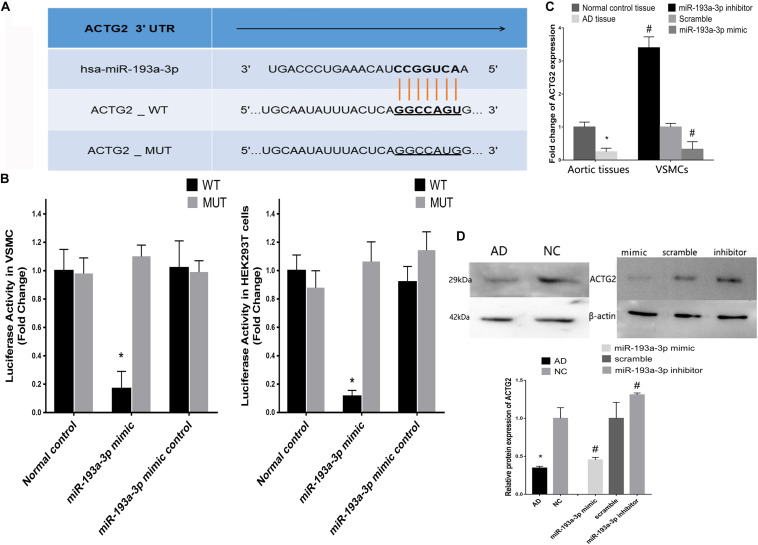
Correlation between miR-193a-3p and ACTG2 in AD. **(A)** miR-193a-3p targets the 3′ untranslated region of ACTG2. **(B)** Dual luciferase assay showing the effect of miR-193a-3p on the wildtype and mutant 3′untranslated region of ACTG2 in HEK293T cells (*n* = 6, *P < 0.05). **(C)** Reduction in the mRNA levels of ACTG2 in tissues from patients with AD and VSMCs transfected with the miR-193a-3p mimic (*P < 0.05, ^#^*P* < 0.05). **(D)** Western blots showing the decrease in the protein levels of ACTG2 in tissues from patients with AD and miR-193a-3p mimic-transfected VSMCs (*P < 0.05, ^#^*P* < 0.05).

Figure 8A shows the predicted binding sites for miR-193a-3p within the 3′ UTR of ACTG2. We used the dual luciferase reporter assay to detect the effect of miR-193a-3p on the wild-type and mutant 3′ UTR of ACTG2. HEK293T cells overexpressing miR-193a-3p decreased luciferase activity to a greater extent in cells expressing wildtype ACTG2, as compared to that in cells transfected with miR-193a-3p control or expressing mutant ACTG2 (Figure 8B). Moreover, qRT-PCR revealed that ACTG2 was downregulated in tissues from AD patients. However, VSMCs transfected with the miR-193a-3p mimic exhibited upregulation of ACTG2 in tissues from healthy individuals and VSMCs depleted of miR-193a-3p (Figures 8C,D).

**FIGURE 9:**
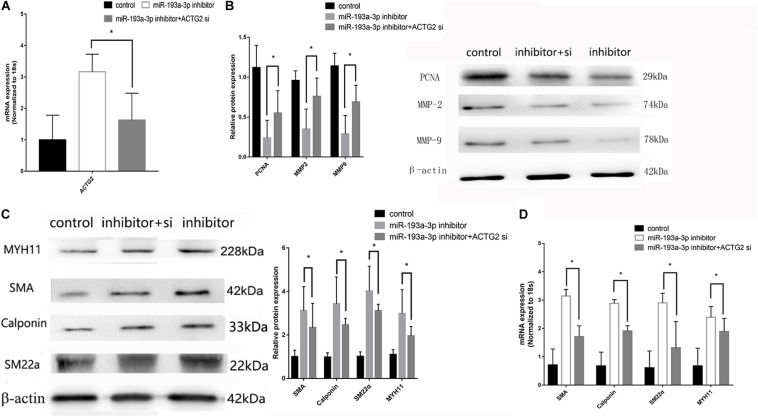
Rescue experiments of ACTG2. **(A)** qRT-PCR presented over/downregulation of ACTG2 after transfection with ACTG2 vector and siRNA, respectively (*n* = 3, *P ≤ 0.05 vs. control). **(B)** Western blot showed remedy PCNA, MMP2, and MMP9 after transfection with miR-193a-3p inhibitor + ACTG2 siRNA than transfection with miR-193a-3p inhibitor only (*n* = 10, * vs. miR-193a-3p inhibitor). **(C)** Western blot analysis showed that low expression of VSMCs differentiation biomarkers in cells transfected with miR-193a-3p inhibitor + ACTG2 siRNA comparing to transfection with miR-193a-3p inhibitor only (*n* = 3, * vs. miR-193a-3p inhibitor). **(D)** qRT-PCR presented remedy expression of VSMC differentiation biomarkers.

pcDNA3.1-ACTG2 vector and ACTG2 siRNA were transfected into VSMC for rescue experiments. qRT-PCR was used to verify the transfection efficiency of pcDNA3.1-ACTG2 vector and ACTG2 siRNA. pcDNA3.1-ACTG2 vector could elevate the expression of ACTG2 in VSMC, whereas ACTG2 siRNA reduced ACTG2 expression (Figure 9A). miR-193a-3p inhibitor + ACTG2 siRNA showed rescue proliferation and migration (Figure 9B) ability compared to that of VSMCs transfected with miR-193a-3p inhibitor only. Phenotypic transition biomarkers involved in VSMC differentiation were lower in miR-193a-3p inhibitor + ACTG2 siRNA than VSMCs transfected with miR-193a-3p inhibitor only (Figures 9C,D).

## Discussion

Vascular smooth muscle cells in aortic media maintain the biological properties of the aortic wall (Rzucidlo et al., 2007; Ren et al., 2017; Chen et al., 2019; Wang et al., 2019b). Proliferation and migration of VSMCs are regulated by phenotypic switching and correlate with the initial stages involved in the development of AD (An et al., 2017a,b; Li et al., 2018; Zhang and Wang, 2019). However, the mechanism(s) of phenotypic switching in VSMCs remain to be understood in detail. In this study, we observed an upregulation of miR-193a-3p in highly proliferative VSMCs and AD tissues. Up- or downregulating miR-193a-3p resulted in the decrease or increase of the differentiation biomarkers (SMA, MYH11, SM22a, and calponin) in VSMCs, respectively. Therefore, miR-193a-3p stimulates cell proliferation and may be a novel modulator of phenotype switching in VSMCs.

In this study, integrating the miRNA and mRNA profiles and using bioinformatics analyses revealed that the miR-193a-3p/ACTG2 axis played an essential role in the pathogenesis of AD. The reason we choose miR-193a-3p/ACTG2 as the target axis is base on the bioinformatic analyses. In addition, PPP1R12B was also predicted as a potential gene in vascular smooth muscle contraction pathway. However, few references reported the interaction between PPP1R12B and vascular smooth muscle contraction pathway as well as AD. miR-193a-3p inhibits the formation, proliferation, and migration of tumor cells in the lung by directly binding to KRAS (Fan et al., 2017). It also functions as a tumor suppressor in colon cancer by interacting with IL17RD (Pekow et al., 2017). However, studies have reported that miR-193a-3p promotes the formation, proliferation, and migration of tumor cells. miR-193a-3p enhances the proliferation and migration of renal carcinoma cells by directly targeting PTEN (Liu et al., 2017). Furthermore, it promotes the development of bladder cancer by targeting HOXC9 (Faugeroux et al., 2015). However, the role of miR-193a-3p on cardiovascular disease, especially AD, has not been studied. Therefore, the identification of miR-193a-3p as a modifier of VSMC phenotype and proliferation will increase our understanding of the pathogenesis of heart disease.

We observed that increased expression of miR-193a-3p (in AD tissues) correlated with the reduced expression of differentiation biomarkers in VSMCs. Furthermore, miR-193a-3p was overexpressed in proliferating human aortic VSMCs that had been proliferating for longer durations. CCK-8 and scrape assays showed that the miR-193a-3p mimic and inhibitor increased and suppressed the proliferation and migration of human VSMCs, respectively. These results help strengthen the inference that miR-193a-3p may be a modulator of phenotypic switching in VSMCs.

MicroRNAs regulate translation by binding to the 3′ UTRs of target mRNAs (Liu et al., 2019). Using various molecular biology techniques and bioinformatics analyses, we have demonstrated that ACTG2 is a target of miR-193a-3p. Overexpression of miR-193a-3p resulted in the downregulation of ACTG2 in transfected VSMCs. These VSMCs exhibited enhanced proliferation and reduced expression of the differentiation biomarkers SMA, MYH11, SM22a, and calponin. In addition, we found the converse regulation between miR-193a-3p and the differentiation markers. So targetscan bioinformatic analyses toold was used to predict binding sites of miR-193a-3p. The results showed no binding sites between miR-193a-3p and SMA, MYH11, SM22a, and calponin. Therefore, we concluded that miR-193a-3p could not directly regulate these differentiation markers.

However, this study also contains severel limitations. All *in vitro* experiments were performed in VSMCs, the different expression of ACTG2 and miR-193a-3p in endothelial remained unclear. In addition, two gene profiles were used in our study, which might cause bias. More sequencing results need to be used for co-expression analysis. Finally, the potential roles of PPP1R12B in VSMCs phenotypic transition need further verification experiments.

In summary, our study revealed that miR-193a-3p was increased in ascending aortic tissues from AD patients. Moreover, miR-193a-3p targeted the 3′ UTR of ACTG2 and may be a novel regulator of phenotypic switching, proliferation, and migration in VSMCs. Thus, the miR-193a-3p/ACTG2 axis may provide mechanistic insight into the pathogenesis of AD and serve as a promising diagnostic biomarker and therapeutic target for AD.

## Data Availability Statement

The datasets presented in this study can be found in online repositories. The names of the repository/repositories and accession number(s) can be found below: GSE98770 and GSE52093.

## Ethics Statement

This study was conducted in accordance with the Declaration of Helsinki and was approved by the Ethical Committee of Jilin University. All patients agreed to participate this program and provided written informed consent.

## Author Contributions

WW designed and supervised the study. TW, KL, HP, YW, and BL performed the analysis work. ZZ and DL contributed to the data analysis. WW and KL organized, designed, and wrote the manuscript. All authors reviewed the final manuscript.

## Conflict of Interest

The authors declare that the research was conducted in the absence of any commercial or financial relationships that could be construed as a potential conflict of interest.

## Footnotes

1 https://genemania.org

2 www.targetscan.org

## Abbreviations

AD: aortic dissection
VSMCs: vascular smooth muscle cells
miRNAs: microRNAs
GEO: gene expression omnibus
GO: gene ontology
KEGG: Kyoto Encyclopedia of Genes and Genomes
DEGs: the differentially expressed genes
CTA: computed tomography angiography
PPI: protein-protein interaction
qRT-PCR: quantitative reverse transcription-PCR
CCK: Cell Counting Kit
FDR: false discovery rate
DEMs: differential expression profile of miRNAs
CAD: coronary heart disease
CC: cellular component
BP: biological processes
MF: molecular function.
